# A Dataset for Detection and Segmentation of Underwater Marine Debris in Shallow Waters

**DOI:** 10.1038/s41597-024-03759-2

**Published:** 2024-08-24

**Authors:** Antun Đuraš, Ben J. Wolf, Athina Ilioudi, Ivana Palunko, Bart De Schutter

**Affiliations:** 1 Authors are with the Laboratory for Intelligent Autonomous Systems (LARIAT), Department of Electrical Engineering and Computing, University of Dubrovnik, Dubrovnik, Croatia; 2 Author is with the Bernoulli Institute, Faculty of Science and Engineering, University of Groningen, Groningen, Netherlands; 3 Authors are with the Delft Center for Systems and Control, Delft University of Technology, Delft, Netherlands

**Keywords:** Computer science, Scientific data

## Abstract

Robust object detection is crucial for automating underwater marine debris collection. While supervised deep learning achieves state-of-the-art performance in discriminative tasks, replicating this success on underwater data is challenging. The generalization of these methods suffers due to a lack of available annotated data considering different sources of variation in the unstructured underwater environment and imaging conditions. In this paper, we present the *Seaclear Marine Debris Dataset*, the first publicly available shallow-water marine debris dataset annotated for instance segmentation/object detection. The dataset contains 8610 images collected using ROVs at multiple locations and with different cameras, annotated for 40 object categories, encompassing not only litter but also observed animals, plants, and robot parts. As part of the technical validation, we provide baseline results for object detection using Faster RCNN and YOLOv6 models. Furthermore, we demonstrate the non-triviality of generalizing the trained model performance to unseen sites and cameras due to domain shift. This underscores the value of the presented dataset in further developing robust models for underwater debris detection.

## Background & Summary

Persistent objects introduced into the marine environment intentionally or unintentionally, as a result of human-induced activities, can be defined as marine debris. Marine debris can injure or even kill marine and coastal wildlife; damage and degrade habitats; interfere with navigational safety; cause economic loss to fishing and maritime industries; degrade the quality of life in coastal communities; and threaten human health and safety^1^. Considering the negative social, economic, and ecological implications associated with pollution, there has been an increase in research dedicated to providing a framework for systematic monitoring and automated collection of marine debris^2^. In this context, two major modalities for detecting marine debris emerged: one focused on surface-floating debris and another dedicated to underwater debris detection. Floating debris detection methods utilize remote sensing technologies such as satellite imagery^3^ and aerial photography^4,5^. To locate underwater marine debris, the development of unmanned vehicles (UxVs) equipped with cameras^6^ and acoustic sensors^7^ is being pursued to detect debris on the seafloor.

Automated underwater marine debris detection from images shares characteristics and challenges of other automatic recognition vision-based tasks in the underwater domain. Geometric and photometric distortions, introduced to the imaging process by the underwater environment, result in numerous quality-diverse data domains^8^. Visual appearance of aquatic scenes can vary drastically based on the conditions such as depth, turbidity, and type of camera sensors used to obtain the imagery. Thus, obtaining diverse data is crucial for the development and evaluation of robust underwater image processing methods.

Supervised learning methods, which achieve state-of-the-art results on discriminative tasks, e.g. object detection, instance segmentation, depend on the availability of annotated data. In the underwater domain such annotated data is sparse compared to the terrestrial domain, which results in an active research area focused on data augmentation^6^ and visual restoration for underwater images^9^.

### Underwater marine debris detection

The only large publicly available repository of underwater marine debris images is the Deep-sea Debris Database (https://www.godac.jamstec.go.jp/dsdebris), curated by the Japan Agency of Marine Earth Science and Technology (JAMSTEC). Available data contains images of marine debris and various types of marine plants and animals captured in underwater surveys by remotely operated vehicles (ROVs), mainly in the sea of Japan. The first work on marine debris detection was done by Fulton *et al*.^10^ who annotated a selection of 5,720 images from the JAMSTEC database and trained the models for the task of plastic debris detection based on four deep learning architectures for object detection - YOLOv2, Faster RCNN, Tiny-YOLO, and Single Shot MultiBox Detector (SSD). The same group of authors extended their previous work by making the *TrashCan* dataset^11^ public, increasing the dataset size to 7,212 images and providing additional annotations for instance segmentation task as well as more detailed classification of debris by material and instance type. In addition, a more consistent and balanced version of the *TrashCan* dataset named UNO^12^ was produced. In this work, the *TrashCan* is further processed to correct wrong label annotations or misplaced bounding boxes, annotating missing objects, and mitigating category imbalance by fusing all trash categories into one category representing all non-natural objects. The key distinction between our dataset and those derived from the Deep-sea Debris Database lies in the data collection environment. Our data was collected in optically shallow waters (i.e. where light reaches the bottom), consequently making the images susceptible to variations in natural light conditions. Another factor that increases the difference between the appearance of objects in deep water with respect to shallow water and deteriorates the visual conditions is the growth of marine biological fouling on underwater objects. This effect, however, rarely takes place in deep water due to the lack of light. Additionally, our dataset includes data from the same sites but captured with different cameras which can be used to test the generalization of models in the presence of cross-camera domain shift. All mentioned datasets are presented in Table 1, along with additional datasets^13,14^ of similar modality (i.e. RGB images taken at close range) containing land litter and floating marine litter.

**Table 1:** Overview of annotated image datasets for underwater marine debris detection.

Dataset	Environment	No. images	Annotation type	No. categories	Year
Trash-ICRA19^26^	Underwater (Plastic, ROV, bio)	5720	Bounding Box	3	2019
TrashCan-Material 1.0^27^	Underwater (Plastic, metal, paper, rubber, wood, etc.)	7212	Mask/Bounding Box	16	2020
TrashCan-Instance 1.0^27^	Underwater (Bag, clothing, rope, wreckage, etc.)	7212	Mask/Bounding Box	22	2020
UNO^12^	Underwater (Bag, clothing, rope, wreckage, etc.)	5902	Bounding Box	4	2022
PlastOPol^28^	Land/floating (Litter)	2418	Bounding Box	1	2022
DeepPlastic^29^	Underwater (Plastic litter)	3200	Bounding Box	1	2021
TACO^14^	Land/floating (Cigarette, plastic film, broken glass, styrofoam piece, etc.)	1500	Mask/Bounding Box	60	2020
CleanSea^30^	Underwater (Litter)	1223	Mask/Bounding Box	19	2022

The main contributions of the current paper are the following:

1. We present *Seaclear Marine Debris Dataset*^15^ first publicly available underwater marine debris dataset in shallow-water environments, annotated for instance segmentation and object detection tasks. The dataset comprises images gathered from various locations, captured using different cameras, thereby creating a multi-domain dataset.
2. As part of the technical validation we provide baseline results for marine debris detection with Faster RCNN and YOLOv6 models.
3. To emphasize the significance of multi-domain data in building robust models, we demonstrate that enhancing objective image quality measures or addressing domain shifts in the input space through image enhancement methods does not improve detection performance or generalization ability in the presence of cross-site or cross-camera domain shifts.

## Methods

This section provides information about the data acquisition setup, including the robots and camera sensors used to collect raw data for *Seaclear Marine Debris Dataset*^15^. Brief descriptions of each site where data collection was performed are given, outlining the human activity in the area and potential sources of pollution. Additionally, we provide a brief summary of the methods used to analyse and demonstrate the non-trivial nature of performing underwater marine debris detection in multi-domain settings.

Degradations introduced by turbidity, floating particles, and the properties of light propagation in water, typically result in images of low visual quality i.e., with color distortion, contrast decrease, and haziness. In addition to camera and medium dependencies, shallow-water images have a strong dependency on natural lighting, which can result in drastically different images for the same site depending on capture time and weather conditions. Low visual quality and domain shift can impair the generalization ability for object detection using deep learning architectures^8^. These issues are typically addressed by image processing techniques categorized as image restoration methods, assuming a known image formation and degradation model, or image enhancement methods that use subjective quality criteria to produce visually pleasing images. Model-based image restoration methods used in underwater environments typically depend on parameters that vary depending on water type, depth, lighting, and camera parameters. The data available within the *Seaclear Marine Debris Dataset* is collected from multiple trials at different sites and captured with cameras of different characteristics. Thus estimating these parameters would be difficult if not impossible without performing a calibration procedure *in situ* for each conducted survey. Since image restoration is not applicable, in this paper we resort to image enhancement techniques that only require a single image as an input.

First, we describe UIQM and Underwater Index, quality assessment metrics used to gain insight into the visual characteristics of each domain. Finally, this is followed by a description of the fusion-based image enhancement method^16^ which we use as a preprocessing step to improve the visual characteristics of the data and reduce the domain shift.

### Data acquisition

Data collection was performed by deploying camera-equipped Remote Operated Vehicles (ROVs) at different sites. A *BlueROV2* was equipped with two cameras, a *Bluerobotics Low-Light HD Camera* and a *Paralenz Vaquita*, while the *SST Mini-Tortuga ROV* was equipped with a *Smart Security SIP-E323CV* camera. Through multiple trials, different lighting and turbidity conditions were encompassed, with each site having a characteristic source of pollution.

- *Portoč (Island of Lokrum), Croatia* - situated 600 meters away from the city of Dubrovnik, is used as a small port for docking of tourist ferries. Data includes footage of debris accumulated mostly as a result of tourist activity together with the vicinity of cruise ship and yacht anchorage. Moreover, some of the images collected from the Lokrum site contain debris that was intentionally placed on the seabed and in the water column by divers for Seaclear project demonstrations (an example can be seen in the LO-II labeled image in Fig. 7). The Lokrum site features clear transparent water and *Posidonia Oceanica* seabeds at 5–10 *m* depth.

**Fig. 1:**
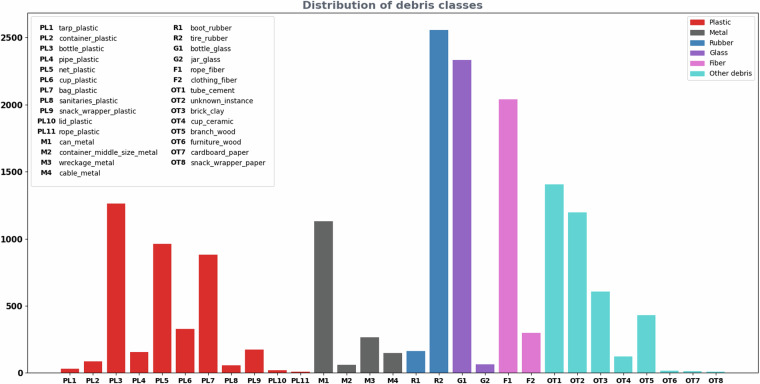
Distribution of debris instances in the dataset images by object categories and materials.

**Fig. 2:**
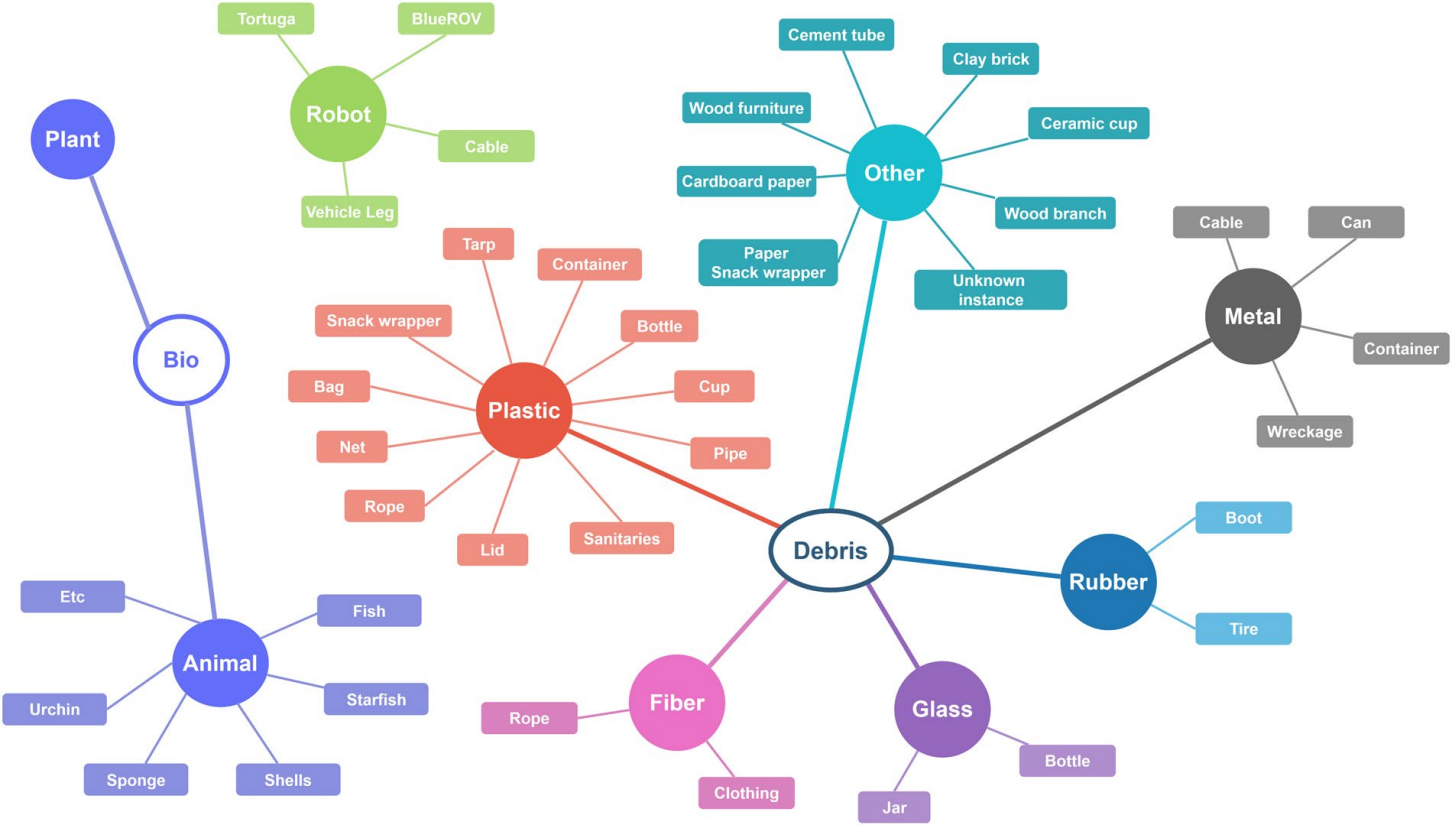
Visualization of the relations between the debris categories and super-categories.

**Fig. 3:**
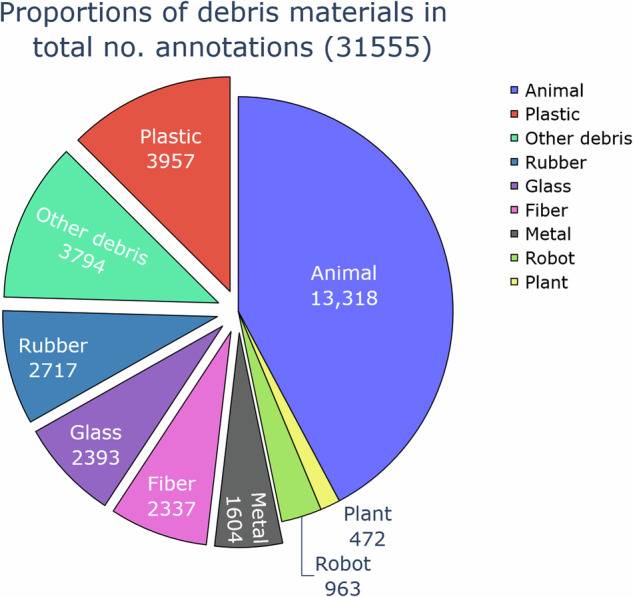
A pie chart showing the proportions of annotated objects belonging to specific debris material, robot, animal and bio categories.

**Fig. 4:**
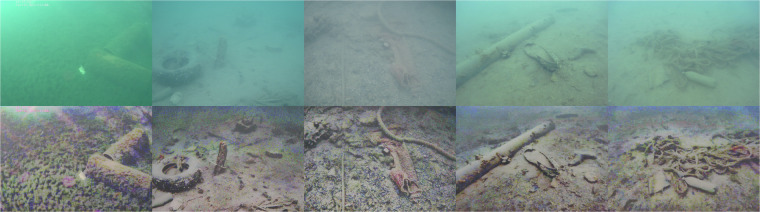
The top row showcases the original images from different data collection sites of our dataset, while the bottom row displays the corresponding Fusion enhanced versions. A more uniform color distribution can be observed in the Fusion-enhanced images.

**Fig. 5:**
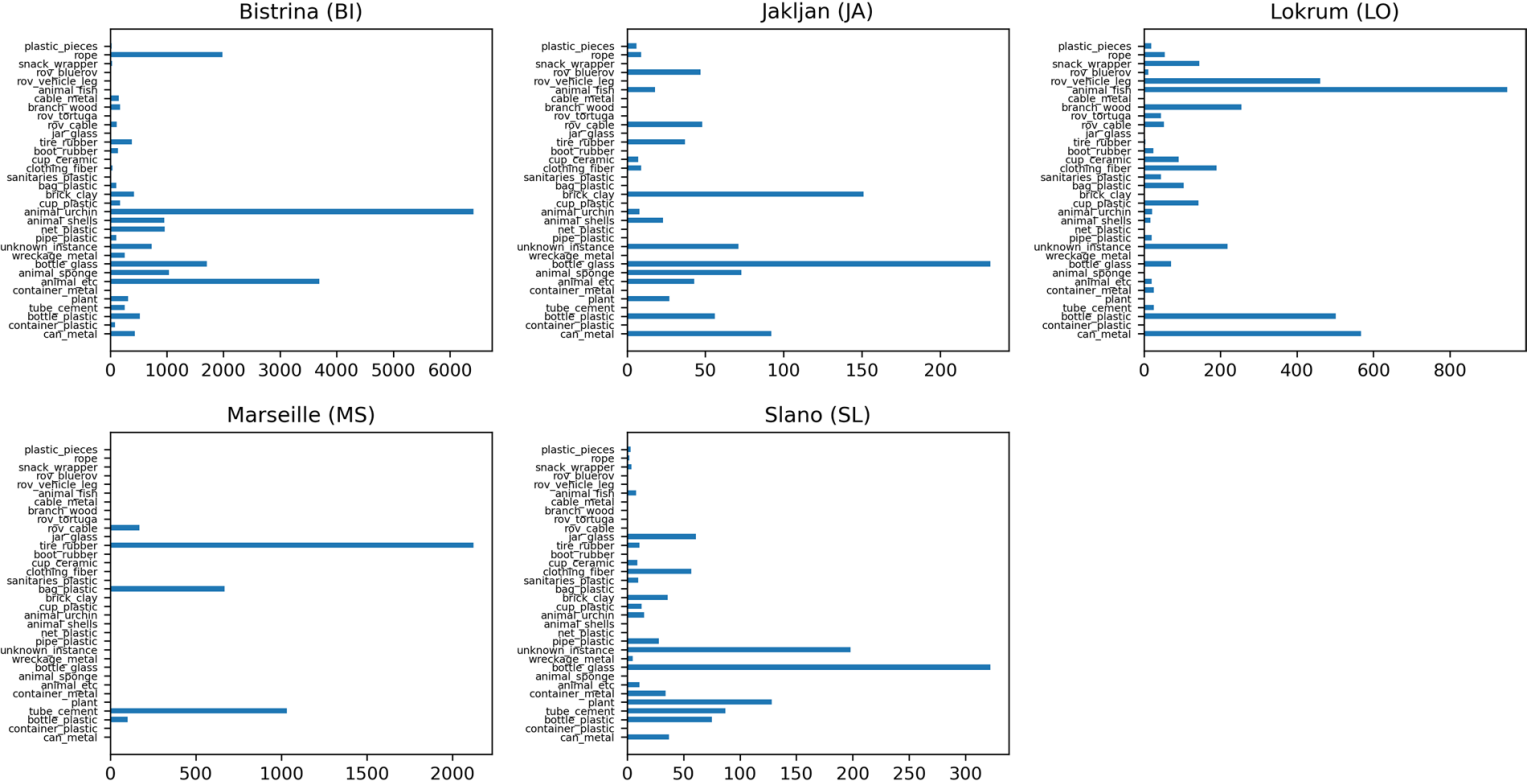
Distribution of object categories for each site used in the evaluation of baseline results.

**Fig. 6:**
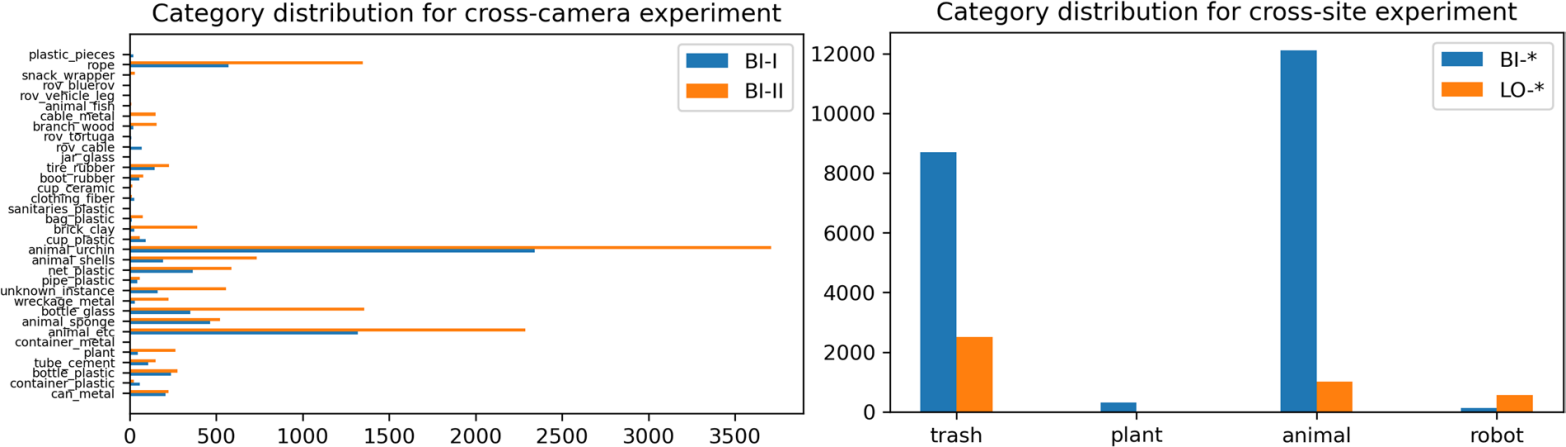
Category distributions for domains used in cross-camera and cross-site experiments.

**Fig. 7:**
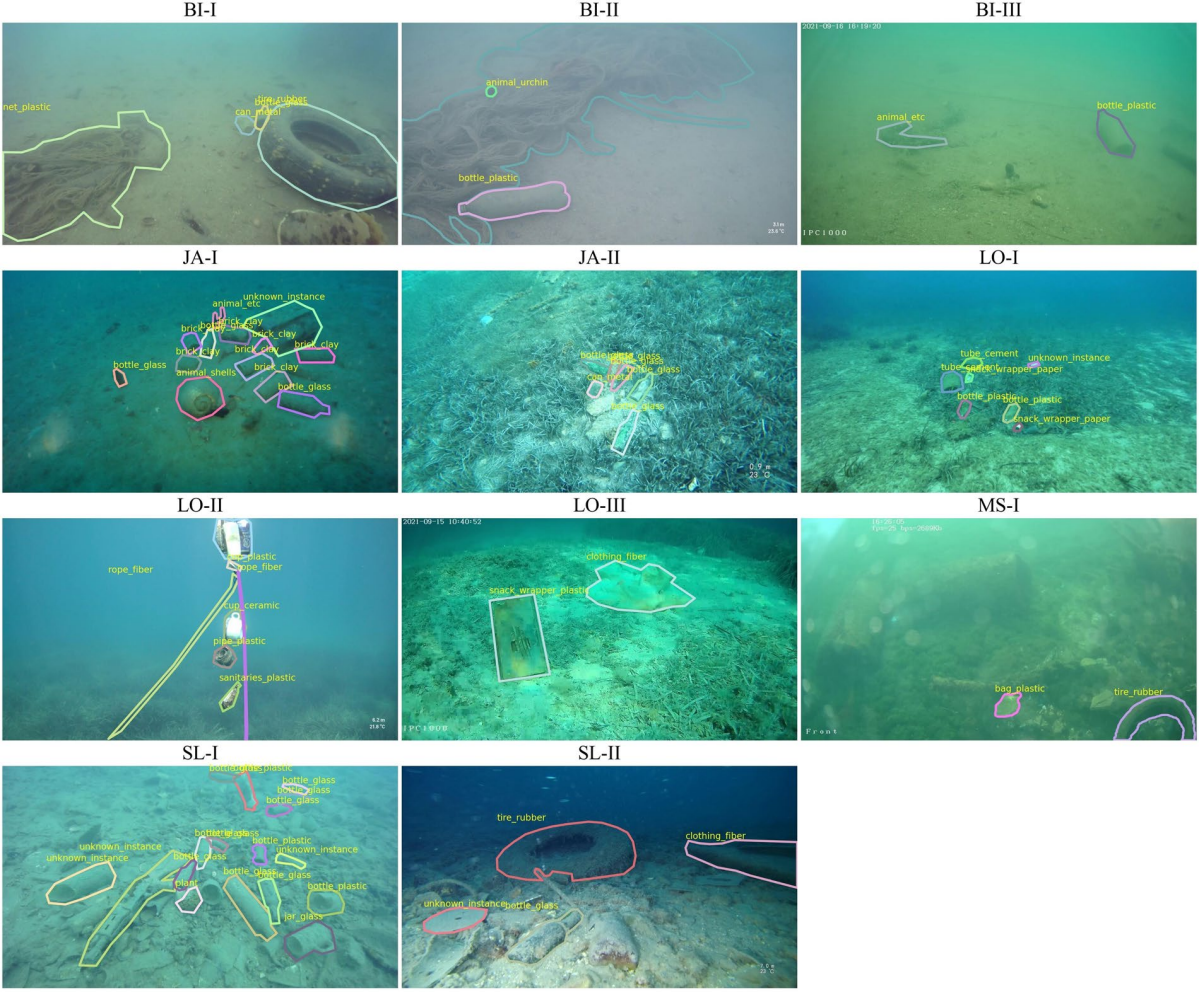
Annotated sample visualization from each domain of the dataset, showing both polygon masks and category labels.

**Fig. 8:**
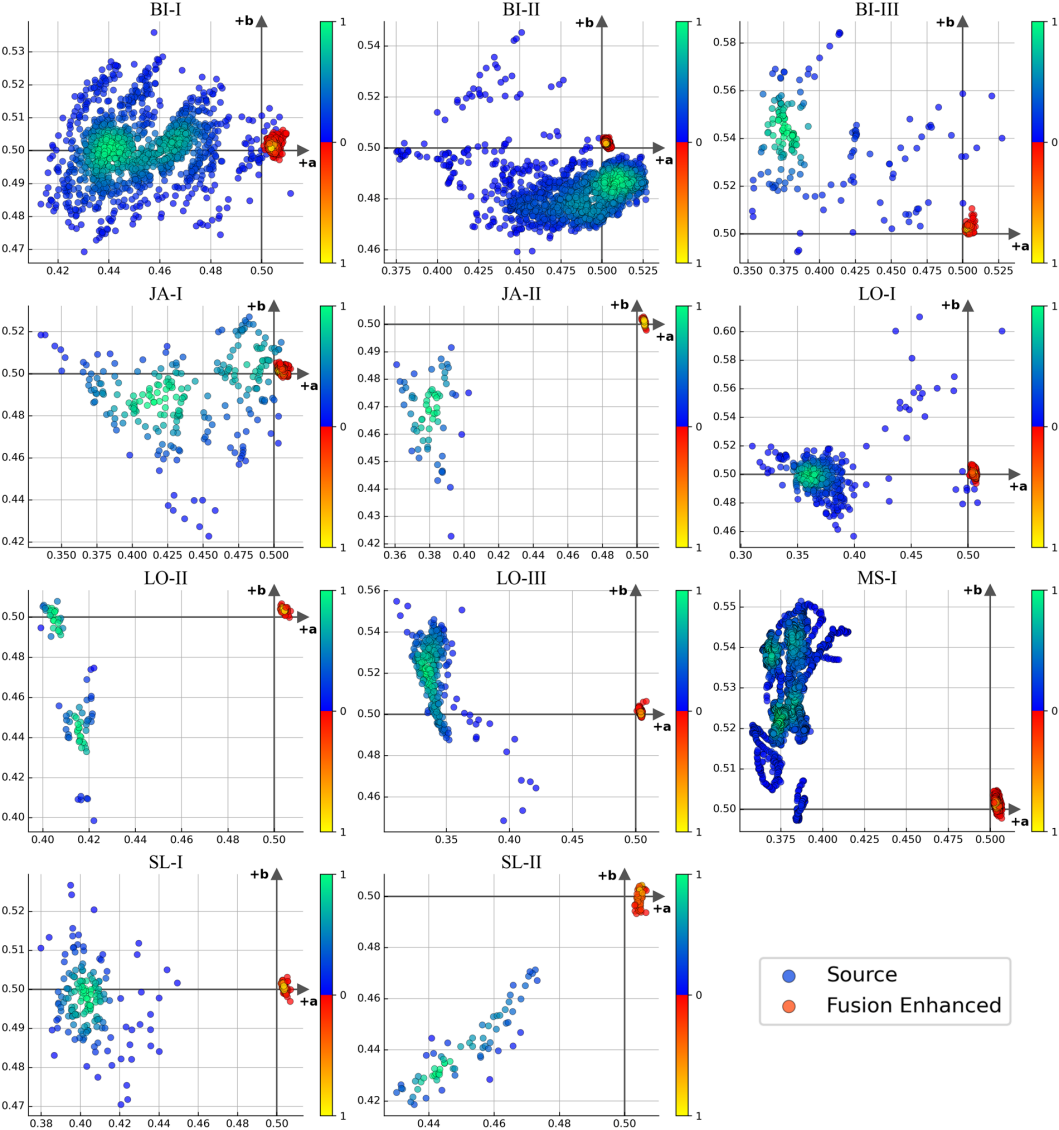
Scatter plots of source and enhanced data per domain in terms of (*a*, *b*) components in Lab color space, showing the distinct grouping of the Fusion-enhanced data around the origin.
- *Bistrina, Croatia* - situated in Mali Ston Bay, the largest production area of the European Flat Oyster (*Ostrea edulis*). Oyster farming activities commonly result in the marine environment being polluted with debris such as plastic shellfish trays, buoys, and nets, which is reflected in the imagery for this site, as seen in Fig. 7. The water is turbid, exhibiting lower visibility due to increased nutrient and sediment concentrations.
- *Slano, Croatia* - a small village with a harbor located 27 km northwest of Dubrovnik. Pollution is mostly the result of harbor and tourist activities along with improper disposal of construction waste. The debris is diverse and clustered, partially covered in construction rubble, which causes it to blend in with the background.
- *L’Estaque (Marseille), France* - suburb area located north of Marseille, in the vicinity of the old port. Most of the debris is the result of the industrial activity and waste dumping.
- *Jakljan, Croatia* - islet belonging to the Elaphites archipelago. The data features a smaller number of individual pieces of debris, mostly bottles and cans as a result of nautical tourism.

### Underwater image quality assessment

The available quality metrics for terrestrial color images are limited in their applicability to underwater images since they fail to consider the extent of degradation and optical properties involved in underwater image formation. Since no reference image is available and subjective measures require time-consuming manual labeling, objective underwater quality measures that aim to capture the objectivity and perception of the human visual system (HVS) are utilized to provide a quality estimation on the *SeaClear Marine Debris Dataset*.

In literature^17^ it was observed on large amounts of underwater image data that the Lab color space has a strong capability of indicating the color distribution and that its (*a*,*b*) components can be used to differentiate between underwater and terrestrial images. Underwater images typically gather further away from the origin, while terrestrial images are usually distributed sparsely around it, which allows formulating the score called Underwater Index^17^ *U* representing the possibility of the image being taken underwater:

1 $$U=\frac{\sqrt{{d}_{o}}}{10\overline{L}{d}_{a}{d}_{b}}$$

where $\overline{L}$ is an average value of the *L* channel, while *d*_*o*_, *d*_*a*_, and *d*_*b*_ are distances from the origin, along the *a* axis, and along the *b* axis, respectively.

UIQM^18^ is composed of UICM, UISM, and UIConM, representing a comprehensive quality of an underwater image, where its sub-indexes evaluate colorfulness, sharpness, and contrast characteristics, respectively.

UICM is calculated in terms of the variance ${\sigma }^{2}$ and the mean μ of the opponent colour components:

2 $${\rm{RG}}=R-G\,{\rm{YB}}=\frac{R+G}{2}-B$$

where asymmetric alpha-trimmed statistics ${\hat{\sigma }}^{2}$ and $\hat{{\rm{\mu }}}$ are used to avoid the effect of outlier intensities on the measure:

3 $${\rm{UICM}}=-\,0.0268\sqrt{{\hat{\sigma }}_{RG}^{2}+{\hat{\sigma }}_{YB}^{2}}+0.1586\sqrt{{\hat{{\rm{\mu }}}}_{RG}+{\hat{{\rm{\mu }}}}_{YB}}$$

To account for the sharpness loss, first an edge image is obtained by applying the Sobel-Feldman operator $S(\,\cdot \,)$^19^ on each color channel of the input image *I*. UISM is then defined as a linear combination based on the enhancement measure estimation function^20^ $\varepsilon (\,\cdot \,)$ measuring the contrast ratio of blocks in each edge image, as follows:

4 $$\begin{array}{c}{\rm{UISM}}=0.299\cdot {\mathscr{E}}(S({{\bf{I}}}_{R}))+0.584\cdot {\mathscr{E}}(S({{\bf{I}}}_{G}))\\ \,\,\,\,+0.114\cdot {\mathscr{E}}(S({{\bf{I}}}_{B}))\end{array}$$

where the weights for each channel are chosen to reflect the response of the human visual system.

The final component accounts for the contrast degradation typically caused by backward scattering using the logAMEE measure on the intensity image. logAMEE combines the logarithmic entropy of the Michelson Contrast and PLIP operators ($\otimes$, $\oplus$, ⊖), which provide nonlinear representation consistent with HVS:

5 $${\rm{UIConM}}=\frac{1}{L\cdot M}\otimes \mathop{\sum }\limits_{l\mathrm{=1}}^{L}\mathop{\sum }\limits_{m\mathrm{=1}}^{M}{\mathscr{M}}({W}_{l,m})\,\mathrm{ln}({\mathscr{M}}({W}_{l,m}))$$

where $L\cdot M$ is the number of blocks and ${\mathscr{M}}(\,\cdot \,)$ is the PLIP Michelson Contrast^21^.

The final value of the UIQM^18^ is given by:

6 $${\rm{UIQM}}={c}_{1}\cdot {\rm{UICM}}+{c}_{2}\cdot {\rm{UISM}}+{c}_{3}\cdot {\rm{UIConM}}$$

where the choice of the coefficients *c*_1_, *c*_2_, and *c*_3_ depends on the application, but generally a higher value of UIQM value corresponds to an image with a better quality.

### Fusion based image enhancement

The Fusion based image enhancement method^16^ is a framework based on blending filters frequently used for image enhancement. This method can be summarized with 3 steps:

1. Obtaining classical enhanced versions of a degraded image
2. Deriving weight maps for each enhanced version
3. Multi-scale fusion using the weight maps

The two derived enhanced versions represent the color-corrected version of the image and the contrast-enhanced version of the underwater image after noise reduction, respectively. These two enhanced versions are referred to as inputs for the Fusion algorithm. The first input is obtained by applying a white balancing technique, which improves the Gray-World illumination estimate by adding a weighting based on the size of the detected set of colors. To remove degradation caused by the scattering in the medium, the second input is derived by applying the local adaptive histogram equalization to a noise-free and color-corrected version of the original image.

Fusion enhancement utilizes 4 weight maps per input to enhance the contrast, saturation, and exposedness of the image. The Laplacian contrast weight (*W*_*L*_) tackles the issue of global contrast by applying a Laplacian filter to each luminance channel of the input and calculating the absolute value of the filter’s output. The local contrast weight (*W*_*LC*_) considers the relationship between each pixel and the average of its neighboring pixels. This measure enhances the visual effect of local contrast by emphasizing transitions, particularly in the highlighted and shadowed areas of the second input. The saliency weight (*W*_*S*_) enhances the visibility of objects that lose their distinctiveness by applying a saliency algorithm based on the biological concept of center-surround contrast. Additionally, to avoid prioritizing highlighted areas in *W*_*S*_ and to protect the mid tones that might be altered in some specific cases, the exposedness weight (*W*_*E*_) is defined. Exploiting the fact that pixels tend to have a higher exposed appearance when their average normalized values are closer to 0.5, *W*_*E*_ is defined as the Gaussian distance to the 0.5 value. This has the effect of tempering the saliency weight and preserving image appearance for non-highlighted areas.

The final enhanced image version is obtained by fusing the defined inputs and weights at multiple scales. Inputs *I*^*k*^ are decomposed into a pyramid by applying the Laplacian operator $L(\,\cdot \,)$ to different scales. The weight maps for the *k*-th input are normalized to satisfy the constraint $\sum {\bar{W}}^{k}=1$. For each normalized weight map $\bar{W}$ a Gaussian pyramid $G(\,\cdot \,)$ is computed. Since both the Laplacian and Gaussian pyramids have the same number of levels *l*, mixing is performed independently for each level. Finally, to compute the enhanced image, we use:

$$E(x,y)=\mathop{\sum }\limits_{k\mathrm{=1}}^{K}{G}^{l}\{{\bar{W}}^{k}(x,y)\}{L}^{l}\{{I}^{k}(x,y)\}$$

Several examples of Fusion enhanced images are shown in Fig. 4.

## Data Records

This section describes the annotated image data in the *Seaclear Marine Debris Dataset*. Taxonomy of debris categories and debris instance distribution are visualized and followed by a brief overview of the dataset’s annotation format and directory structure. The dataset is made publicly available at 4TU.ResearchData repository^15^ under the CC BY 4.0 license.

### Dataset structure

The *Seaclear Marine Debris Dataset* is comprised of 8610 underwater marine debris images, captured utilizing BlueROV and Mini-Tortuga ROVs and annotated for instance segmentation/object detection tasks. Object instances annotated in this dataset can be semantically grouped into 3 super-categories:

- *debris* - objects found in the marine environment as a result of human-induced activities,
- *bio* - marine vegetation and animals,
- *robot* - ROVs used for data collection and their parts.

Debris annotations are categorized by instance type and material, both encoded in the category name as *{instance}*_*{material}*. The taxonomy of the class categories is depicted in Fig. 2, while the distribution of the debris categories included in the dataset is shown in Fig. 1. For the *bio* category, the animal species was indicated in the annotations, while for the *robot* category the model of the ROV and specific robot parts such as cable or vehicle leg which frequently appeared in the images, were indicated. Proportions of specific debris materials in the total number of annotations are shown in Fig. 3.

Images were labeled using *labelme*^22^, a tool that allows creating polygon annotations for instance segmentation and that provides easy conversion from the *labelme* JSON format to the frequently used VOC and COCO formats. The dataset directory structure reflects the site at which data was captured and the camera used to obtain the images as follows:
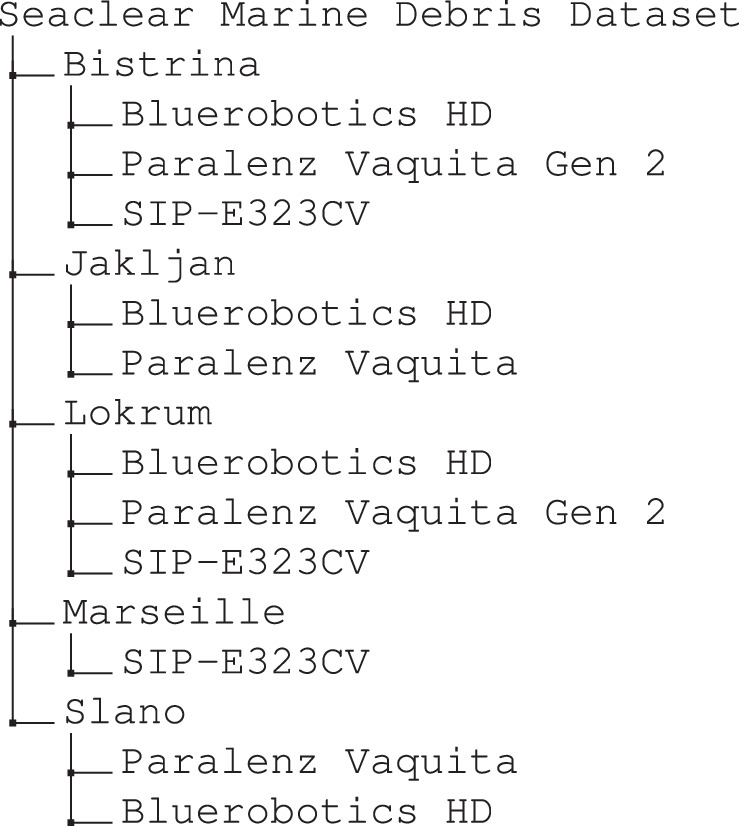


Information on the number of images and annotations for each sub-folder are provided in Table 2. Visualized samples of annotated images from the dataset can be seen in Fig. 7.

**Table 2:** No. images per dataset domains, grouped by data collection sites and cameras.

Site	Camera	Domain Label	No. images
Bistrina,Croatia	Bluerobotics Low-Light	BIS-I	1390
	Paralenz Vaquita Gen 2	BIS-II	2069
	SIP-E323CV	BIS-III	193
Jakljan,Croatia	Bluerobotics Low-Light	JA-I	241
	Paralenz Vaquita	JA-II	65
Lokrum,Croatia	Bluerobotics Low-Light	LO-I	556
	Paralenz Vaquita Gen 2	LO-II	77
	SIP-E323CV	LO-III	339
Marseille,France	SIP-E323CV	MS-I	3441
Slano,Croatia	Bluerobotics Low-Light	SL-I	168
	Paralenz Vaquita	SL-II	71
		**Total**	**8610**

## Technical Validation

To enable new research and to provide better insight into the characteristics of the provided data and open challenges, the following experiments were conducted as part of this work:

1. Baseline results on combined data from all sites in *Seaclear Marine Debris Dataset*^15^ for the debris detection task are given with both a two-stage Faster RCNN and a one-stage YOLOv6 detector to provide a reference for future work.
2. Analysis of domain effect on object detection performance in shallow-water imagery is conducted based on cross-camera and cross-site data splits. By comparing performance on source and fusion-enhanced data we investigate the effects of image enhancement on improving the generalization ability of trained models.

The results are summarized in Table 4 and Table 5. The experiments and the results are preceded by a brief overview of the architectures and design choices for Faster RCNN and YOLO V6 models used to conduct the experiments as part of the technical validation.

### Baseline models

For obtaining the baseline results on the *SeaClear Marine Debris Dataset* for marine debris detection task we used the Faster RCNN and YOLOv6^23^ models, which we briefly review in this section. Faster RCNN^24^ is a two-stage model and more computationally expensive; on the other hand, YOLOv6 is a lightweight model suited for real-time inference.

#### FasterRCNN

The Faster RCNN architecture can be divided into the following sub-modules:

- **Backbone Network** is a CNN used to extract features from the input image (e.g. ResNet, VGG, AlexNet), typically pre-trained on a large dataset such as ImageNet. The choice of the backbone model determines the number of model parameters and the representation ability of the model.
- **Region Proposal Network (RPN)**: This is the first stage of the model that generates a set of region proposals, or regions of interest (RoIs), which are image areas likely to contain objects. The RPN is a lightweight CNN that is trained to predict the objectness score and to regress the offsets for a fixed number of anchor boxes at each spatial location.
- **RoI Pooling** is applied to the feature maps generated by the backbone network, it converts the generated RoIs into fixed-size feature maps, which can then be fed into the second stage of the model.
- **Fast R-CNN Head**: The second stage of the model consists of fully-connected layers that produce the final output by predicting class probabilities and bounding box coordinates.

#### YOLOv6

As in the Faster RCNN architecture, YOLOv6 uses a CNN backbone network to extract features from the input image; however, since YOLOv6 is a single-stage detector, it predicts the class probabilities and the bounding box without explicitly generating regions of interest. Single-stage detectors generally consist of two additional submodules: a neck and a head. The neck of the network is used for aggregation of low-level spatial features and high-level semantic features, which are used by the head to produce the final detection results. Design choices for the YOLOv6 submodules are made aiming to improve the computational efficiency and accuracy trade-off when compared to its predecessors, specifically YOLOv5 and YOLOX models.

The EfficientRep backbone used in YOLOv6 utilizes the re-parametrization strategy inspired by RepVGG^25^ to decouple the multi-branch topology at learning time and to provide a simpler single path topology at inference time. The Rep-PAN neck is a modified version of the PAN topology from YOLOv4 and YOLOv5 models that replaces the CSP-Block with a RepBlock for small models and CSPStackRep for large models. Unlike YOLOv5 the design of the YOLOv6 detection head decouples the classification and regression layers like YOLOX, but reduces the number of convolution layers to increase efficiency.

### Experimental setup

Baseline results were obtained using a conventional approach to object detection. A two-stage Faster RCNN and a one-stage YOLOv6 model initialized with COCO dataset weights were finetuned to the *Seaclear Marine Debris Dataset*. We used a larger (41.6 M parameters) FasterRCNN model, utilizing a Resnet-50 backbone with FPN (Feature Pyramid Network) for feature extraction and a smaller YOLOV6 S model (17.2 M parameters).

Faster RCNN training was performed for 150 epochs using the cosine annealing (with warm restarts) learning policy with a base learning rate of ${\lambda }_{0}\,=\,0.001$ and ${T}_{0}=10$ steps, with a multiplication factor of 2. Reported results were obtained using $640\times 360$ input images and mini-batches of size 2. For YOLOv6 S finetuning was done based on the implementation available in the official repository. The model was fine-tuned for 400 epochs with a batch size of 32 and an input image size of $640$ $\times$ $360$.

To mitigate the impact of categories with a small number of annotations we consolidated categories in the dataset that had fewer than 50 annotations by grouping them with appropriate, related categories. For example, *snack_wrapper_plastic* (8 annotations) and *snack_wrapper_paper* (172 annotations) were combined into *snack_wrapper* (180 annotations) category. This process resulted in the final dataset of 34 categories used to conduct the experiments presented in further text.

### Baseline results

To obtain the training and test set for the baseline evaluation we performed an 80%-20% random split on the whole dataset. Both models exhibited high performance in this setting with YOLOv6 S outperforming the Faster RCNN model by ≈7%. This is most likely due to better regularization of the smaller model on a dataset of this size and YOLOv6 using multiple data augmentation strategies, which were not utilized on Faster RCNN. Performance was consistent for both models across sites, as seen in Table 4, with both models showing the highest performance on the Marseille data, most likely due to the lowest inter-category variance (as seen in Fig. 5) and the static nature in which the data was collected. Also, both models exhibit the lowest performance on the Slano data, which is the most challenging site in our dataset due to the small sample size, various types of debris, and partial occlusion of objects by construction rubble causing them to blend with the background.

Additionally, we evaluated both models on baseline data split with fusion-enhanced images. For Faster RCNN, we report a 2.7% performance drop in terms of mAP, compared to evaluation on unprocessed data; however, on YOLOV6, a slight improvement of 0.5% was achieved.

### Effects of image enhancement and evaluating generalization

As seen in Table 3 processing the images with Fusion-based image enhancement seems to provide a two-fold benefit by significantly improving the image quality as measured by UIQM and by lowering the Underwater Index. Visualizing the enhanced images versus the source images in terms of (*a*,*b*) components of Lab color space (see Fig. 8), shows that this sort of processing eliminates the color distribution gap between different domains in the image input space. However, these improvements in terms of objective quality metrics do not correspond to consistent improvement of object detection performance nor a better generalization ability of the model. In a cross-site setting, both models perform poorly and no performance improvements are observed from applying image enhancement.

**Table 3:** Per domain UIQM and Underwater Index values, for source and fusion enhanced data.

	UIQM		Underwater Index	
	Source	Fusion Enhanced	Source	Fusion Enhanced
BIS-I	1.544	3.358 ↑	13.932	2.246 ↓
BIS-II	1.978	3.507 ↑	22.672	2.122 ↓
BIS-III	1.898	3.212 ↑	6.106	2.085 ↓
JA-I	2.692	3.426 ↑	5.024	2.941 ↓
JA-II	2.252	3.490 ↑	2.376	1.949 ↓
LO-I	2.279	3.274 ↑	2.768	3.104 ↑
LO-II	1.991	3.443 ↑	4.777	1.496 ↓
LO-III	1.598	3.136 ↑	3.496	3.558 ↑
MS-I	1.606	3.291 ↑	7.345	1.878 ↓
SL-I	2.928	3.416 ↑	8.209	4.762 ↓
SL-II	2.084	3.447 ↑	3.985	2.192 ↓

Evaluating in a cross-camera setting, where the model was trained on BIS-I data and evaluated on BIS-II data we observed an improvement of 2.1% in terms of mAP. However, this improvement does not hold for the inverse case where the model is trained on the BIS-II data and evaluated on BIS-I data. It is important to note that for these two domains, the shift was caused only by camera pose offset and camera type since both were mounted on the same ROV during the data collection survey. This results in a minimal difference in terms of category distribution between training and validation data as seen in Fig. 6. However, there is still a significant drop in performance compared to the baseline results where data from all domains is readily available. This suggests that a significant part of the domain shift can be attributed to using different cameras and the changes in point of view from how each camera was mounted.

In a cross-site setting, imbalanced category distributions between training and validation data are unavoidable because the marine life and type/quantity of debris depend on specific locations and human activity. This issue is exaggerated by the large number of categories as objects are less likely to belong to the same category. Using the full 34 categories of the dataset was not viable in the cross-site setting as there was little overlap between the category distributions. In an attempt to produce a more balanced distribution, two strategies were explored: aggregating debris categories based on materials (plastic, metal, rubber, fiber, glass, and other debris) as previously done in literature^10^ and consolidating all debris categories into a single category. Non-debris categories were grouped into one of the appropriate categories: *robot*, *animal* or *plant*. Data from the Bistrina site is used as training data because it provides the most diverse domain. Lokrum site was chosen for validation data because it has data available from the same set of cameras as the training domain, allowing us to isolate the site effect on the domain shift and evaluate its impact on performance. As demonstrated in Table 4, the performance in terms of mAP degrades further in cross-site scenarios. This outcome is expected, as using different sites for training and validation significantly affects the variance in category distribution and increases intra-category variance. An outlier among the cross-site experiments is the top-performing one, reporting a mAP of $\mathrm{20.4 \% }$. Upon inspecting the AP per category, it was observed that this result was driven by the model’s high average precision (AP) for the *robot* category and not by improved debris detection performance.

**Table 4:** Stratified split baseline results.

Model	Fusion Enhancement	mAP (%)	mAR(%)	mAP(%) per site				
				Bistrina	Jakljan	Lokrum	Marseille	Slano
Faster RCNN R50 + FPN		61.7	68.1	60.7	58.2	55.9	80.4	32.6
Faster RCNN R50 + FPN	✓	59.0	65.6	57.2	51.9	52.4	79.2	28.7
YOLOv6 S		68.3	75.0	69.5	66.7	59.3	79.0	46.9
YOLOv6 S	✓	**68.9**	**75.4**	69.6	70.2	60.3	80.6	46.2

We report the mean Average Precision (mAP) in Table 5 using the full 34 categories of the dataset for cross-camera experiments and using the 4 aggregated categories for cross-site experiments. Object category distributions for both sets of generalization experiments can be seen in Fig. 6.

**Table 5:** Evaluation of generalization performance in cross-camera and cross-site setting.

Cross-camera Domain Generalization					
Model	Fusion Enhancement	Split		mAP (%)	mAR (%)
		Train	Test		
Faster RCNN R50 + FPN		BIS-II	BIS-I	14.5	22.0
Faster RCNN R50 + FPN	✓	BIS-II	BIS-I	14.21	23.45
YOLOv6 S		BIS-II	BIS-I	**26.2**	**46.6**
YOLOv6 S	✓	BIS-II	BIS-I	24.4	44.5
Faster RCNN R50 + FPN		BIS-I	BIS-II	12.0	18.0
Faster RCNN R50 + FPN	✓	BIS-I	BIS-II	14.16	21.41
YOLOv6 S		BIS-I	BIS-II	24.7	38.8
YOLOv6 S	✓	BIS-I	BIS-II	**26.0**	**40.4**
**Cross-site Domain Generalization**					
Faster RCNN R50 + FPN		{BIS-*}	{LO-*}	5.0	9.7
Faster RCNN R50 + FPN	✓	{BIS-*}	{LO-*}	3.7	8.3
YOLOv6 S		{BIS-*}	{LO-*}	**20.4**	**51.1**
YOLOv6 S	✓	{BIS-*}	{LO-*}	7.2	42.7

## Usage Notes

Annotations and additional information about the dataset are stored in COCO (Common Objects In Context) style format (single JSON file). Using this standard annotations format allows utilizing open-source tools, like COCO API (https://github.com/cocodataset/cocoapi) for easy access through multiple programming languages (Matlab, Lua, Python). More complex utility functions for visualizing and modifying annotations are available in open-source Python packages, such as KWCOCO (https://github.com/Kitware/kwcoco).

## Data Availability

The custom code used for generating figures, conducting image quality analysis, and image enhancement can be found in the repository associated with this publication (https://github.com/adjuras/seaclear-dataset). Our MATLAB code used for Fusion based image enhancement is a slightly modified version of an open-source implementation available online (https://github.com/fergaletto/Color-Balance-and-fusion-for-underwater-image-enhancementr.-). Official UIQM implementation used to obtain values presented in Table 3 is available for download per request on the author’s website (https://karen-panetta.squarespace.com/download). YOLOv6 S implementation used for the technical validation is available in the official repository (https://github.com/meituan/YOLOv6).
